# The Use of Blocking Wires in Fibular Intramedullary Nailing of an Ankle Fracture with Hardware Failure: A Case Report

**DOI:** 10.7759/cureus.47737

**Published:** 2023-10-26

**Authors:** Daniel Acevedo, Michael G Rizzo, David Constantinescu, Marilyn Heng

**Affiliations:** 1 Orthopaedic Surgery, Nova Southeastern University Dr. Kiran C. Patel College of Allopathic Medicine, Davie, USA; 2 Orthopaedic Surgery, University of Miami Miller School of Medicine, Jackson Memorial Hospital, Miami, USA

**Keywords:** intramedullary nail, revision ankle surgery, fibula fracture, ankle fracture management, syndesmotic injury, syndesmotic disruption, im nail

## Abstract

A 24-year-old male, with a body mass index (BMI) of 31.7 and a previous open reduction and internal fixation (ORIF) of the left ankle seven years ago, presented to the emergency department with a peri-implant, comminuted fibula fracture with broken hardware and syndesmotic injury. The nature of the revision surgery made proper guidewire placement during fibular nailing difficult. Blocking wires assisted in ensuring proper guidewire placement. The patient was successfully managed with revision ORIF, fibular nailing, and syndesmotic fixation. Blocking wires are a helpful tool for achieving proper fracture alignment and stability during intramedullary nailing procedures and may be considered in fibular nailing situations.

## Introduction

Lateral malleolus/distal fibula fractures with syndesmotic injury are common sports-related injuries that typically occur after a rapid twisting motion [1]. This type of injury often requires surgical treatment to stabilize the ankle joint, restore normal joint kinematics, and restore function [2,3]. Failure to properly identify and reduce a syndesmotic injury can lead to chronic joint instability, osteoarthritis, and significant morbidity [1]. Lateral malleolus fractures have historically been treated with plate and screw fixation through an open lateral approach [4]. This necessitates open reduction and visualization of the fibula and extent of fracture. Intramedullary nailing is an increasingly used technique for treating these fractures, offering the advantages of smaller incisions for open reduction and nail insertion as well as less soft tissue dissection and preservation of periosteal blood supply, at the price of increased implant cost [4-6]. However, certain circumstances, such as comminuted fractures or revision surgeries with previous hardware and screw tracts, can cause the guidewire to deviate resulting in an inability to pass the guidewire or malreduction of the fibula and syndesmosis. One helpful technique in such cases is the use of poller-blocking screws and wires, which provide additional stability and guide the path of the guidewire or nail [7-9]. The term "poller" originates from the German word "poller," which means "post" or "bollard," and was first described by Krettek et al. in 1999 as a means to control the alignment during intramedullary nailing of long bone fractures [9]. The blocking wire or screw prevents deviation of the guidewire, reamer, and implant into undesired locations. As such, blocking technique can be a useful skill in a surgeon's armamentarium to assist in fracture reduction and implant position.

Here, we present a patient with a syndesmotic injury and a peri-implant, comminuted fibula fracture with broken hardware managed with revision open reduction and internal fixation (ORIF), fibular nailing, and syndesmotic fixation. We will discuss the pearls of the technique, including precautions for blocking wire handling and the use of flexible cannulation.

The patient provided consent after being informed that data concerning their case would be submitted for publication.

## Case presentation

The patient, a 24-year-old male, with a body mass index (BMI) of 31.7, no significant past medical history, and a previous ORIF of the left ankle in 2016 presented to the emergency department after sustaining a new left ankle fracture while playing soccer. Notably, the previous left ankle fracture was also from a fall while playing soccer, which was successfully treated with ORIF at a hospital in Venezuela. The patient recovered well from his first fracture and denied any history of ankle pain or instability prior to either injury.

On physical examination, the left lower extremity showed significant swelling with ecchymoses of the medial aspect of the ankle. The skin was grossly intact, and the patient was exquisitely tender to palpation over the medial and lateral malleoli. Leg compartments were soft and compressible, but the patient was unable to range his ankle due to pain and stiffness. Sensation was intact to light touch across all nerve distributions, and the patient was able to fire relevant muscle groups. Palpable pulses and brisk capillary refill were present.

**Figure 1:**
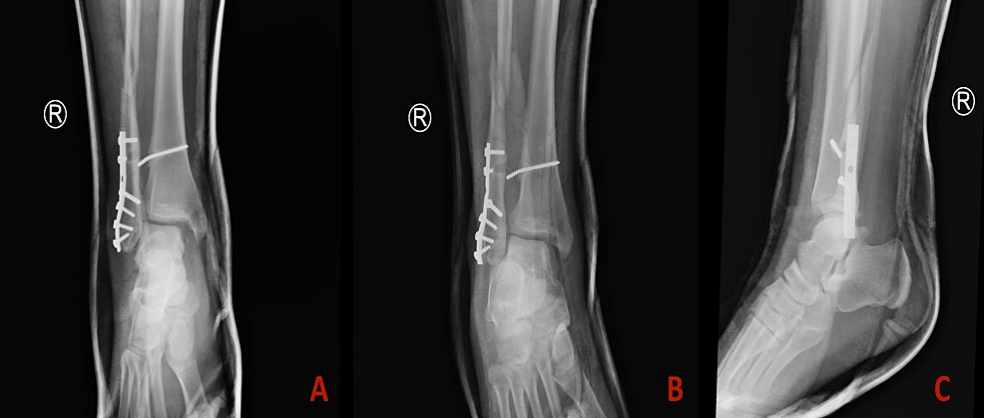
Preoperative radiographs from 05/04/2023 depict fibular fracture and previous ORIF hardware. A: Anterior-posterior view. B: Oblique view. C: Lateral view ORIF: open reduction and internal fixation

Preoperative radiographs (05/04/2023) (Figure 1) revealed a previous ORIF of the lateral malleolus with 1/3 tubular plate and cortical screws as well as a broken syndesmotic screw, with a bent tip causing syndesmotic disruption. Additionally, there was a fracture of the fibula above the level of the plate with multifragmentary comminution. The patient was evaluated by the orthopedic surgery team, and a decision was made to proceed with surgical intervention to restore ankle stability.

Surgical planning aimed to restore the length, alignment, and rotation of the fibula by choosing a fixation method that sufficiently spanned the fracture while promoting biological conditions for fracture healing. We ultimately decided to proceed with a revision ORIF using a fibular intramedullary nail and syndesmotic fixation. The fibular nail permitted spanning of the fracture with a longer implant which required less incision and muscle dissection than a plate of similar length would have. There is also an added benefit in that the implant could be augmented for increased syndesmotic fixation by placing screws or suture through holes within the nail.

After discussion of all treatment options, the patient elected to undergo the proposed treatment with removal of hardware and revision fixation. The patient was taken to the operating room and placed under general anesthesia. After prepping and draping the left leg in the standard fashion, a lateral incision was made over the fibula to expose the previous hardware. The broken syndesmotic screw and existing ORIF hardware were removed. The shaft of the broken syndesmotic screw was removed by approaching it anterior to the fibula in the syndesmotic space.

**Figure 2:**
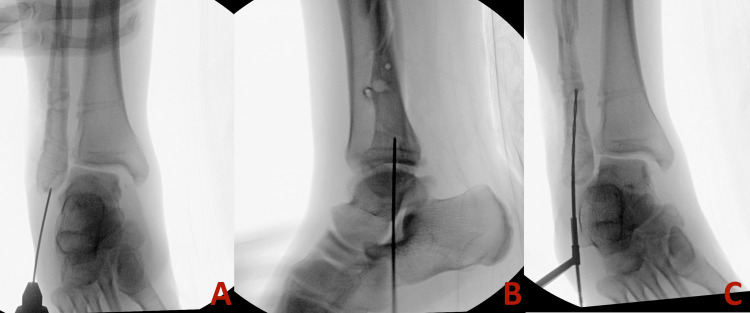
Intraoperative radiographs depicting guidewire placement impinged on medial fibula cortex preventing further advancement of the guidewire. A: Anterior-posterior view. B: Lateral view. C: Oblique view

**Figure 3:**
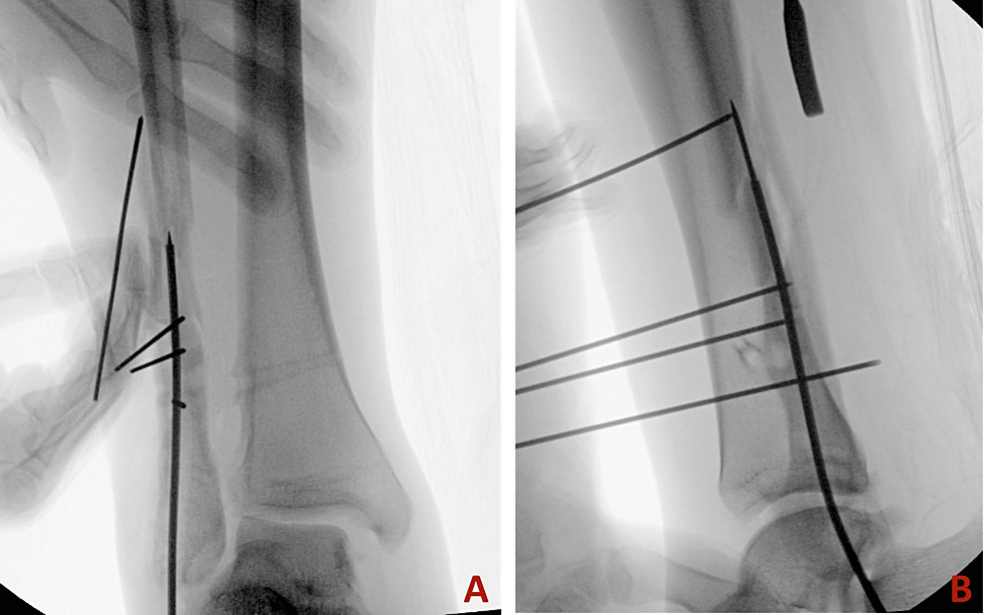
Intraoperative radiographs depicting the use of blocking wires to prevent medial deviation. A: Anterior-posterior view. B: Lateral view

**Figure 4:**
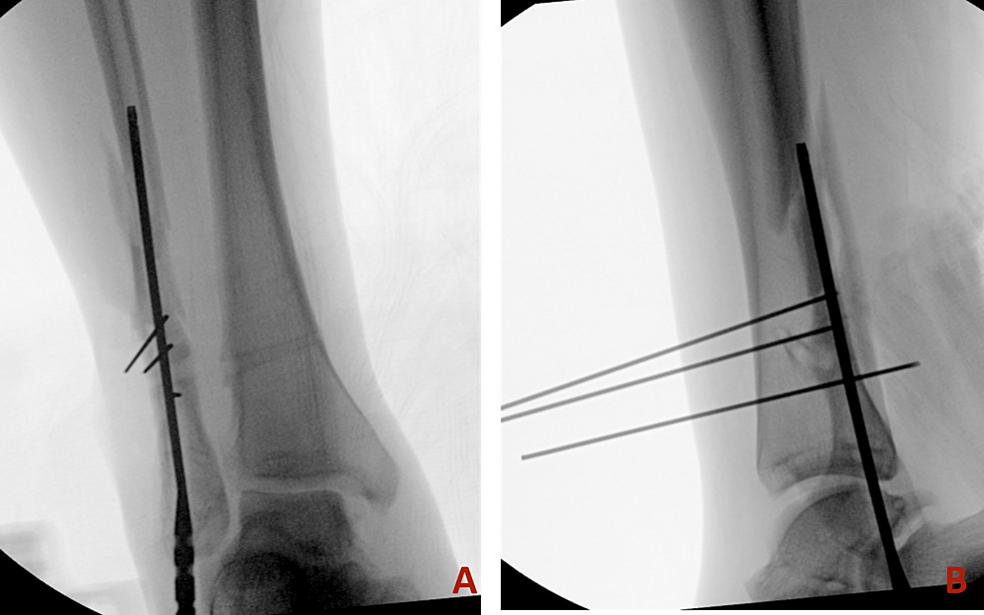
Intraoperative radiographs depicting the advancement of the fibular nail over the guidewire past the blocking wires. A: Anterior-posterior view. B: Lateral view

The guidewire was inserted from the tip of the fibula into the canal. The presence of previous screw tracts and associated intramedullary sclerosis prevented smooth guidewire advancement, causing medial deviation through the comminution of the fracture (Figure 2). We attempted using a solid drill bit to correct the path, but it alone was not successful. To facilitate passage, three 1.6 mm K-wires were placed sequentially to guide the path of the guidewire and prevent deviation. With the guidance of these three blocking wires and a more rigid cannulated "finger," the guidewire was advanced beyond the fracture into the proximal segment (Figure 3 and Figure 4).

**Figure 5:**
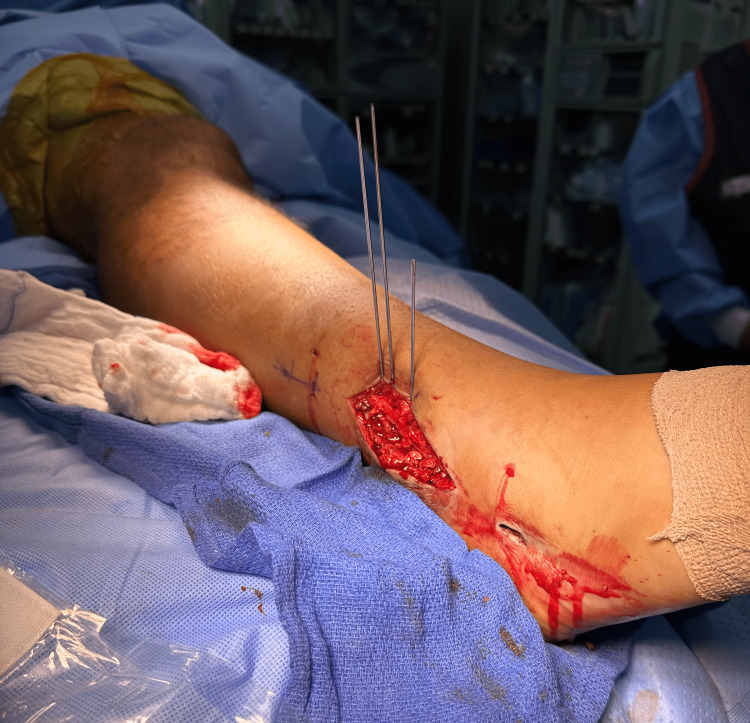
Intraoperative picture depicting the placement of blocking wires through the larger incision used for the removal of hardware. The more distal, smaller incision was used for reaming and for nail insertion

The intramedullary canal was then sequentially reamed to the appropriate diameter, and the fibular nail (FibuLock®, Arthrex, Naples, Florida, United States) was inserted over the guidewire (Figure 5). Actuation of the FibuLock® talons, which provide proximal rotational control, and placement of distal locking screws secured the nail in position. During the removal of the blocking wires after the distal locking of the nail, one of the wires broke due to the significant stress placed across it from reaming and nail passage because of its small caliber. However, the wire was left slightly proud posteriorly, allowing for retrieval using a needle driver after dissecting posterior to the fibula.

**Figure 6:**
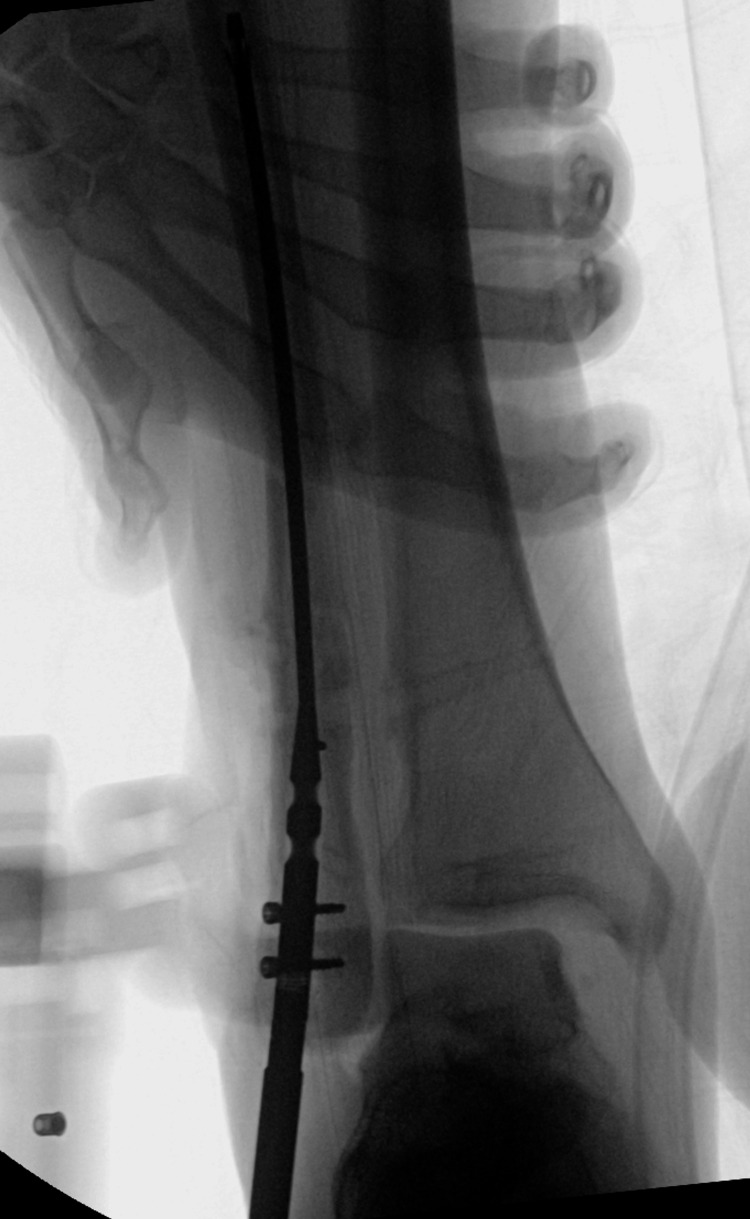
Intraoperative radiograph performing an external stress depicting medial clear space widening as well as talar lateral shift indicative of syndesmotic instability

**Figure 7:**
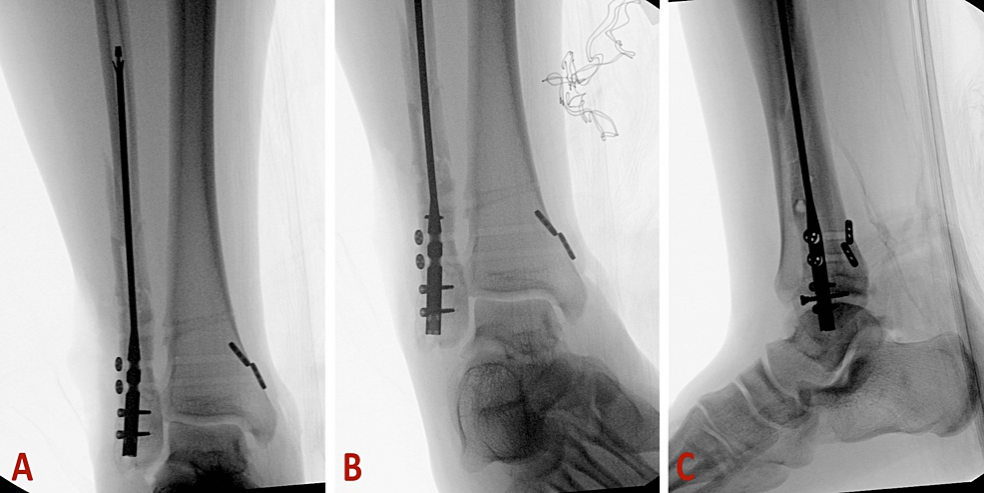
Intraoperative radiographs depicting placement of the two suture constructs through the fibular nail. A: Anterior-posterior view. B: Oblique view. C: Lateral view

Syndesmotic stability was assessed using dorsiflexion and external rotation stress. Under live fluoroscopy, there were lateral translation of the talus and widening of the syndesmosis indicating instability (Figure 6). The decision was made to proceed with syndesmotic fixation using two tensionable suture constructs (TightRope®, Arthrex, Naples, Florida, United States). The medial gutter was opened, and scar tissue was removed to allow for reduction of the syndesmosis using a Weber clamp. The suture constructs were then drilled and placed through the nail, tensioned appropriately, and secured (Figure 7).

The wounds were irrigated and closed in the standard fashion, and a sterile dressing was applied. The patient was placed in a splint with toe-touch weight-bearing restrictions and transferred to the postoperative care unit for recovery.

**Figure 8:**
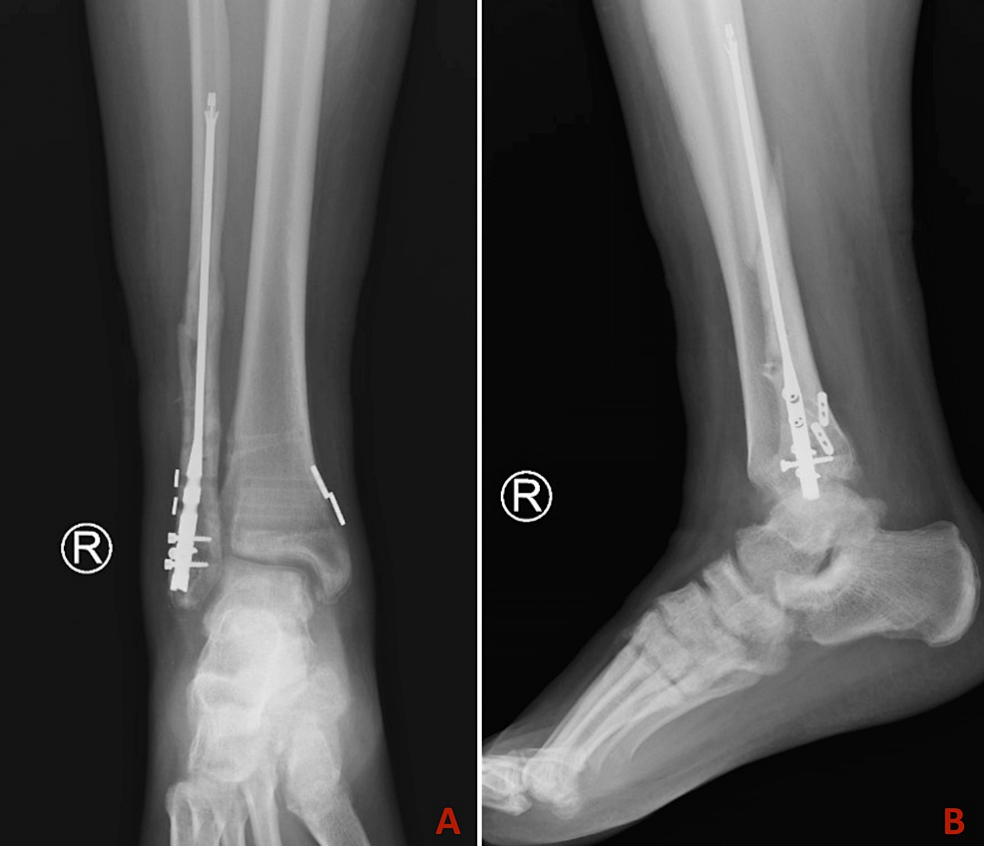
Postoperative radiographs at three-month follow-up. A: Anterior-posterior view. B: Lateral view

The patient presented to the clinic for follow-up at two weeks and at three months postoperatively. At the three-month follow-up, the patient was successfully weight-bearing without pain or symptoms of instability and is working with physical therapy on strengthening and returning to full activity. Examination of the right ankle revealed a fully healed surgical wound with no erythema/dehiscence/drainage. The patient was neurovascularly intact, including the superficial peroneal nerve. The patient reported being satisfied with the surgical outcome and had no complaints. Postoperative radiographs from the three-month follow-up can be seen in Figure 8.

## Discussion

The prevalence and detrimental effects of ankle fractures have been well documented in the literature, roughly accounting for 9% of all orthopedic injuries [10-12]. Their postoperative period after an open reduction and internal fixation is typically complicated by pain, stiffness, and instability of the ankle joint [13]. Many patients may experience unsatisfactory outcomes, with hardware prominence being a common complaint which requires implant removal [14,15]. Therefore, the decision-making process in treating the left ankle fracture of this 24-year-old patient underlines the importance of careful preoperative planning and an individualized approach in selecting the most suitable treatment option. The existing hardware, comminuted fracture, and syndesmotic dislocation presented unique challenges that required a thorough evaluation of available treatment options.

Revision to a long fibular plate was considered but deemed less favorable due to the proximal extension of the fracture and the need for extensive muscle dissection, compared to a fibular intramedullary nail which has similar union rates and functional outcomes yet fewer wound complications and implant removals [16,17].

The use of blocking wires is an extremely useful technique to assist in obtaining the appropriate nail path and alignment of the fracture during an intramedullary implant procedure. When encountering challenges during the insertion of the guidewire, blocking wires prove essential in correcting the wire's trajectory and ensuring accurate fracture reduction. By strategically placing blocking wires adjacent to the fracture site, orthopedic surgeons can guide the guidewire along the intended path, promoting proper fracture alignment and even increasing the biomechanical stability of the final construct if replaced with retained blocking screws. This technique significantly reduces the risk of complications related to malalignment and improper reduction, enhancing the success rate of the procedure and minimizing the occurrence of postoperative issues such as nonunion or implant failure [18,19]. The decision to include a screw for blocking versus a wire alone with removal at the completion of the case is an important consideration. In instances where fracture stability is a concern or optimal alignment is challenging to achieve, the inclusion of a screw for blocking can provide added stability. In the case of our patient, the nail was noted to have excellent cortical interference and stable alignment of the fracture following wire removal, so we felt there was no need for screw insertion. Additionally, with the small size of the fibula, there are limited space for screw insertion and a high risk of iatrogenic fracture.

## Conclusions

Herein, we demonstrate an interesting case of hardware failure and syndesmotic injury treated with intramedullary fibular nail and revision syndesmotic fixation. The intramedullary nail, combined with the use of blocking wires, proved to be an appropriate choice in this case. The unique aspects of this 24-year-old patient with existing hardware, comminuted fracture, and syndesmotic dislocation underscore the value of a tailored approach to decision-making in orthopedic surgery. Furthermore, it highlights the usefulness of blocking wires in obtaining the appropriate nail path and alignment of the fracture during intramedullary nailing procedures.
